# The Role of the Hydrogen Bond Network in Maintaining Heme Pocket Stability and Protein Function Specificity of *C. diphtheriae* Coproheme Decarboxylase

**DOI:** 10.3390/biom13020235

**Published:** 2023-01-25

**Authors:** Federico Sebastiani, Chiara Baroni, Gaurav Patil, Andrea Dali, Maurizio Becucci, Stefan Hofbauer, Giulietta Smulevich

**Affiliations:** 1 Dipartimento di Chimica “Ugo Schiff”, DICUS, Università di Firenze, Via della Lastruccia 3-13, I-50019 Sesto Fiorentino, Italy; 2 Department of Chemistry, Institute of Biochemistry, University of Natural Resources and Life Sciences, Muthgasse 18, A-1190 Vienna, Austria; 3 INSTM Research Unit of Firenze, via della Lastruccia 3, I-50019 Sesto Fiorentino, Italy

**Keywords:** heme biosynthesis pathway, resonance Raman spectroscopy, propionate H-bond strength, bending modes, decarboxylation

## Abstract

Monoderm bacteria accumulate heme *b* via the coproporphyrin-dependent biosynthesis pathway. In the final step, in the presence of two molecules of H_2_O_2_, the propionate groups of coproheme at positions 2 and 4 are decarboxylated to form vinyl groups by coproheme decarboxylase (ChdC), in a stepwise process. Decarboxylation of propionate 2 produces an intermediate that rotates by 90° inside the protein pocket, bringing propionate 4 near the catalytic tyrosine, to allow the second decarboxylation step. The active site of ChdCs is stabilized by an extensive H-bond network involving water molecules, specific amino acid residues, and the propionate groups of the porphyrin. To evaluate the role of these H-bonds in the pocket stability and enzyme functionality, we characterized, via resonance Raman and electronic absorption spectroscopies, single and double mutants of the actinobacterial pathogen *Corynebacterium diphtheriae* ChdC complexed with coproheme and heme *b*. The selective elimination of the H-bond interactions between propionates 2, 4, 6, and 7 and the polar residues of the pocket allowed us to establish the role of each H-bond in the catalytic reaction and to follow the changes in the interactions from the substrate to the product.

## 1. Introduction

**Figure 1:**
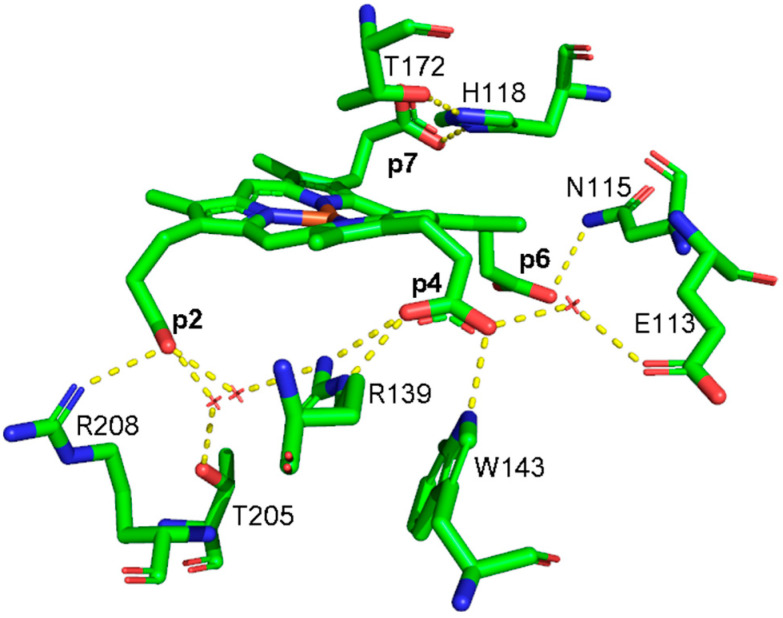
Structure of the active sites of coproheme complex of *Cd*ChdC WT (PDB ID: 6XUC), showing the hydrogen bonds (yellow dotted lines) between the polar residues and the propionate groups as observed in Chain A [13]. Red crosses indicate water molecules.

In 2015, the coproporphyrin-dependent heme biosynthesis (CPD) pathway utilized by monoderm bacteria (Firmicutes and Actinobacteria) to produce heme *b* was discovered [1,2,3,4]. The coproheme decarboxylase (ChdC) in the presence of two molecules of H_2_O_2_ generates heme *b* by a two-step decarboxylation of the propionate groups of coproheme at positions 2 (p2) and 4 (p4), forming vinyl groups (Figure S1). After the cleavage of p2, the transiently formed monovinyl monopropionyl deuteroheme (MMD) reaction intermediate [5,6,7,8] rotates by 90° inside the protein pocket, bringing p4 near the catalytic tyrosine [9,10,11,12], to allow its decarboxylation and finally to form heme *b*. In common with other structures of ChdC from different bacteria, namely *Listeria monocytogenes* (*Lm*ChdC) (PDB ID: 6FXQ, 6FXJ) [9] and *Geobacillus stearothermophilus* (PDB ID: 5T2K) [7], *Corynebacterium diphtheriae* ChdC (*Cd*ChdC) (PDB ID: 6XUC) [13] has a homopentameric structure, and each subunit has the active binding site for the substrate in its C-terminal domain. The active site of the wild-type (WT) protein is characterized by a series of amino acid residues that provide a binding site for coproheme and, at the end of the reaction, for heme *b*. The stabilization of the substrate within the protein pocket comprises an extensive hydrogen bond network involving water molecules, specific amino acid residues, and the propionate groups of the porphyrin ring in addition to a proximal histidine-heme iron bond. According to the crystal structure, each propionate group of coproheme is involved in one or more hydrogen bonds (H-bonds) with the surrounding polar amino acids (Figure 1, Table 1) [13]. The propionate group p2 is involved in three hydrogen bonds with R208, T205, and R139. Among those, only the R208 residue is directly bound to the propionate, while the remaining two amino acids interact through a water molecule. Furthermore, the R139 residue is also directly H-bonded to the propionate group p4. This latter propionate is also bound to two other amino acid residues: directly to W143 and via a water molecule to E113. The propionate group in position 7 (p7) is hydrogen-bonded directly to the distal H118 residue, which is in turn H-bonded to T172, while the propionate group in position 6 (p6) is hydrogen-bonded to N115.

However, the orientation of the substrate within the active site varies during the reaction process since, following the decarboxylation of p2, the reaction intermediate MMD rotates clockwise by 90° inside the protein pocket [11,12], thus inducing a reorganization of the propionate interactions within the cavity.

The heme peripheral substituents have been implicated in a wide variety of proteins (e.g., cytochromes, peroxidases and globins) as key components in controlling the biochemical properties, including stability and orientation of the substrate upon binding, modulation of reactivity and functionality [14,15,16,17,18,19,20]. In order to evaluate the role of the H-bonds of the porphyrin propionate side chains in the pocket stability and enzyme functionality of *Cd*ChdC, we produced and spectroscopically characterized single and double variants in which H-bonding interactions to propionates 2, 4, 6, and 7 were specifically eliminated. The results allowed us to establish the role of the H-bonds in the stabilization of the coproheme inside the active site before decarboxylation, and their effect on the catalytic reaction to produce heme *b*. In addition, we were able to evaluate the strength of the interactions before and after decarboxylation, upon selective identification of the vibrational bending modes of the porphyrin propionates by resonance Raman (RR) spectroscopy.

## 2. Materials and Methods

### 2.1. Generation, Expression and Purification of CdChdC and Variants

Site-directed mutagenesis, apoprotein expression and purification of *Cd*ChdC for the WT protein and variants, as well as heme *b* reconstitution have been described previously [10,11,13,21]. Site-directed mutagenesis was performed using the QuikChangeLightning (Agilent Technologies) kit. Primers used to generate variants are listed in Table S1. For singly mutated variants, *Cd*ChdC WT plasmid was used as the template, and for double variants, each respective single variant was used as a template.

### 2.2. Sample Preparation

The *Cd*ChdC coproheme complexes were prepared by adding a coproheme solution in 0.5 M NaOH to *Cd*ChdC apoprotein diluted in 50 mM phosphate buffer, pH 7.0 in a 2:1 apoprotein:coproheme ratio. The *Cd*ChdC coproheme complexes were eventually titrated to heme *b* complexes by progressively adding small aliquots of a 1 mM H_2_O_2_ stock solution in 50 mM phosphate buffer, pH 7.0, for all of the investigated variants, as already detailed in [11].The complete heme *b* formation was followed by recording the electronic absorption spectra of the variants upon addition of two equivalents (eqs.) of H_2_O_2_, while for the wild type protein, the heme *b* complex was obtained by hemin reconstitution [10] to avoid the presence of additional iron chlorin-type heme *d* species [22] or residual MMD species. The concentration range was between 25 and 35 µM for all of the samples, and determined as described previously [11].

### 2.3. Electronic Absorption Measurements

Electronic absorption measurements were performed in a 5 mm NMR tube or a 1 mm cuvette at room temperature by a Cary60 spectrophotometer (Agilent, Santa Clara, CA, USA) with a resolution of 1.5 nm and a 300 nm/min scan rate.

The second-derivative spectra were calculated by applying the Savitzky–Golay method on 15 data points with a third-order polynomial (LabCalc; Galactic Industries, Salem, NH, USA), after verifying that no changes in the wavelength or in the bandwidth were observed when the number of points was increased or decreased. All of the spectra were normalized to the maximum intensity of the Soret band.

### 2.4. Resonance Raman Measurements

The resonance Raman (RR) spectra were obtained at room temperature under similar excitation conditions using either 406.7 nm excitation of a Kr^+^ laser (Coherent, Innova300 C, Coherent, Santa Clara, CA, USA) for the WT and H118A/H118F variants, as reported in Refs. [10,11], or the 404.8 nm line of a diode laser (MatchBox Series, Integrated Optics, Vilnius, Lithuania) for all other investigated variants. Backscattered light from a slowly rotating 5 mm NMR tube was collected and focused into a triple spectrometer (ActonResearch, Acton, MA, USA), consisting of two SpectraPro 2300i instruments working in subtractive mode and a SpectraPro 2500i instrument in the final stage with a grating of 3600 grooves/mm (with a resolution of 1.2 cm^−1^) and equipped with a liquid nitrogen-cooled CCD detector. The RR spectra were calibrated with indene and carbon tetrachloride as standards to an accuracy of 1 cm^−1^ for intense isolated bands. The power on the samples was 3.5 and 4.5 mW for the coproheme and heme *b* complexes, respectively.

All RR measurements were repeated several times under the same conditions to improve the signal-to-noise ratio, and the spectra were summed only if no spectral differences were noted, as reported in Table S2. The RR spectra were baseline-corrected and normalized in the high wavenumber region to the intensity of the ν_4_ band, at 1370–1377 cm^−1^, and in the low wavenumber region to the intensity of the ν_8_ band, at 341–350 cm^−1^.

The curve-fitting analysis of the spectra was performed by using a spectral simulation program (LabCalc; Galactic Industries, Salem, NH) with a Voight line shape (with a 20% Lorentzian contribution) to determine the peak wavenumbers, bandwidth (full width at half maximum) and intensities, with an accuracy of 1 cm^−1^ for the peak positions and the bandwidths. The use of a Voigt line shape allowed us to account for both lifetime and inhomogeneous line broadening. The latter derived from the differences between the different subunits in the homopentameric structure of the protein (see Table 1 and Ref. [13]). Since most of the bands overlapped in the RR spectra, the fitting procedure with unconstrained parameters was performed iteratively: starting from a run with all free parameters, the widths of the bands corresponding to the same vibrational modes (e.g., the propionate bending modes) were progressively fixed to the same value for all samples. As a further check, the widths of the less-defined bands were fixed alternatively and were shown to give the same final results.

## 3. Results

### 3.1. Coproheme Complexes

**Figure 2:**
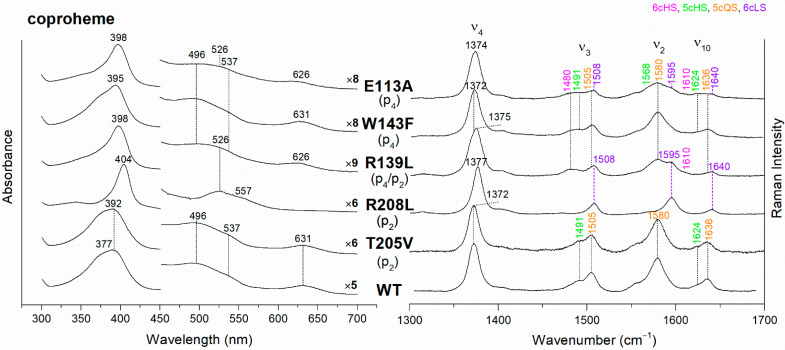
(**Left**) UV-vis electronic absorption spectra, and (**Right**) RR spectra in the high wavenumber region of coproheme complexes of *Cd*ChdC WT and variants. In brackets, the propionate, whose corresponding H-bond is removed by mutation, is reported. The RR core-size marker band wavenumbers are reported in pink, green, orange and violet for the 6cHS, 5cHS, 5cQS and 6cLS species, respectively. In black, at the side of the UV-vis spectra, are indicated the expansion factors applied in the 450–700 nm spectral range for better data visualization.

**Figure 3:**
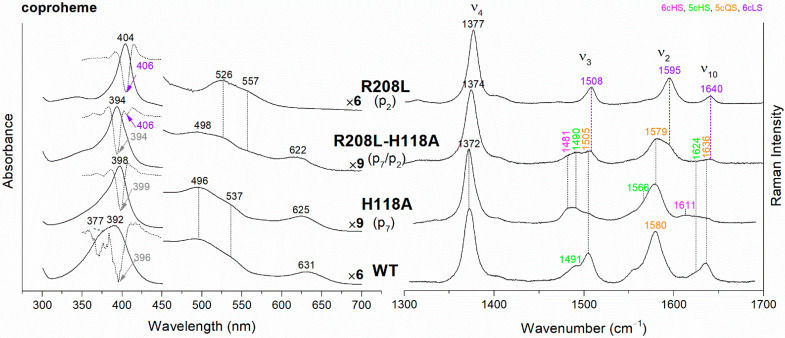
(**Left**) UV-vis electronic absorption spectra (solid lines) and their second derivative (dashed lines), and (**Right**) RR spectra in the high wavenumber region of coproheme complexes of *Cd*ChdC WT and variant complexes. In brackets, the propionate, whose corresponding H-bond is removed by mutation, is reported. The band wavelengths assigned to the 6cLS species are indicated in violet. The RR core-size marker band wavenumbers are reported in pink, green, orange and violet for the 6cHS, 5cHS, 5cQS and 6cLS species, respectively. In black, at the side of the UV-vis spectra, are indicated the expansion factors applied in the 450–700 nm spectral range for better data visualization.

The UV-vis and RR spectra of the coproheme complexes of *Cd*ChdC WT and variants, whose mutation involves the hydrogen bonds with p2 and p4, are compared in Figure 2. The WT protein is a mixture of five-coordinated (5c) quantum mixed-spin (QS) and high-spin (HS) species [11]. The mutation of the T205 (p2) with valine does not give rise to any change in the spectra as compared to WT, suggesting that the T205-p2 H-bond observed in the crystal is not maintained in solution. On the contrary, mutation of R208 (p2) to leucine (Figure 2 and Figure 3) dramatically alters the porphyrin pocket, since the protein becomes a pure hexacoordinated (6c) bis-histidine (His-Fe-His) low-spin (LS) species (Soret band at 404 nm with its second derivative at 406 nm, Q bands at 526 and 557, absence of the CT1 band, and RR core size marker bands ν_3_, ν_2_, and ν_10_ at 1508, 1595, and 1640 cm^−1^, respectively). In fact, the sixth ligand has been identified with the distal H118, since in the double mutant R208L-H118A, the spectra (Figure 3) show an increased contribution of an aquo 6cHS form, typical of the H118A variant [11], with respect to the WT, and only a minor residual 6cLS species, in agreement with the band at 406 nm, is observed in the UV-vis second derivative spectra. The formation of a low-spin species is also found in both the R139L (p2/p4) and E113A (p4) variants (Figure 2), as indicated by the red shift of the Soret band to 398 nm in the UV-vis spectra and the presence of the RR bands assigned to ν_3_, ν_2_, and ν_10_ at 1508, 1595, and 1640 cm^−1^, respectively, together with the formation of an aquo 6cHS species (ν_3_ and ν_10_ at 1480 and 1610 cm^−1^, respectively). On the contrary, the W143F mutation (p4) induces only minimal variations in the spectra, showing a minor 6cHS species in addition to the 5cQS and 5cHS forms (Figure 2).

Unlike the H-bonds with p2 and p4, the lack of interaction with p6 does not significantly affect the active site (Figure S2). In fact, we have studied the N115R and N115L variants, but neither the breakage (N115L) nor the strengthening (N115R) of the H-bonds with the p6 induce any clear change in the spectra, as compared to the WT, except for the appearance of a minor 6cHS population. As concerns the p7 H-bond interaction, we have previously reported that the H118F variant is very similar to the WT, while the H118A mutation caused the formation of a prevalent aquo 6cHS species (Figure S2 and Ref. [11]). Interestingly, the alteration of the H-bond between the T172 and H118 gives rise to spectra almost identical to those of H118A (Figure S2).

### 3.2. Titration of the Coproheme Complexes with Hydrogen Peroxide

**Figure 4:**
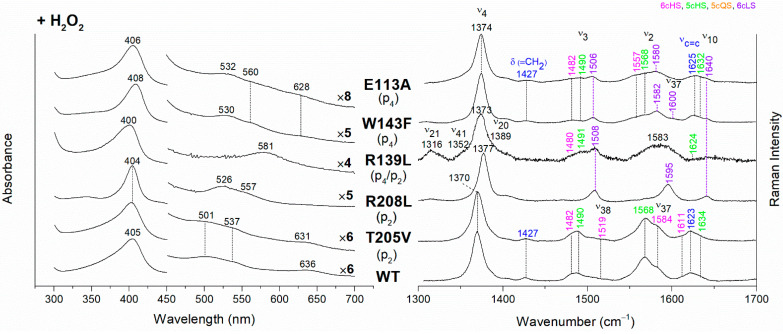
(**Left**) UV-vis electronic absorption spectra and (**Right**) RR spectra in the high wavenumber region of coproheme complexes upon H_2_O_2_ titration of *Cd*ChdC WT and variants. In brackets, the propionate, whose corresponding H-bond is removed by mutation, is reported. The RR core-size marker band wavenumbers are reported in pink, green, orange and violet for the 6cHS, 5cHS, 5cQS and 6cLS species, respectively, while in blue are indicated the vinyl vibrations (ν_C=C_ stretching modes and δ(=CH_2_) bending modes), and in black, at the side of the UV-vis spectra, are indicated the expansion factors applied in the 450–700 nm spectral range for better data visualization.

The *Cd*ChdC heme *b* complexes were obtained from their corresponding coproheme complexes by H_2_O_2_ titration (see Materials and Methods for details). The hydrogen peroxide-titrated UV-vis and RR spectra of coproheme–*Cd*ChdC complexes of the variants lacking the H-bond with p2 and p4, are reported in Figure 4 together with those of the WT heme *b* complex. The electronic absorption spectrum of heme *b*–*Cd*ChdC WT complex shows a Soret band at 405 nm, Q bands at 501 and 537 nm, and the CT1 band at 636 nm. The red-shift of the Soret and Q bands towards longer wavelengths, as compared to the coproheme complex, is due to the conjugation of the double bonds of the vinyl groups (which are formed as a result of the decarboxylation) with the double bonds of the tetrapyrrole ring. Accordingly, the RR spectrum is typical of a mixture of 6cHS and 5cHS species with the presence of the vinyl vibrations at 1623 cm^−1^ (ν_C=C_ stretching modes) and 1427 cm^−1^ (δ(=CH_2_) bending modes) [10,11].

**Figure 5:**
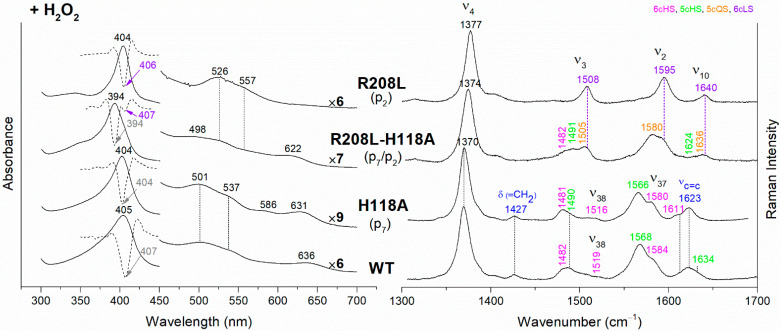
(**Left**) UV-vis electronic absorption spectra (solid lines) and their second derivative (dashed lines), and (**Right**) RR spectra in the high wavenumber region of coproheme complexes upon H_2_O_2_ titration of *Cd*ChdC WT and variants. In brackets, the propionate, whose corresponding H-bond is removed by mutation, is reported. The band wavelengths assigned to the 6cLS species are indicated in violet. The RR core-size marker band wavenumbers are reported in pink, green, orange and violet for the 6cHS, 5cHS, 5cQS and 6cLS species, respectively, while in blue are indicated the vinyl vibrations (ν_C=C_ stretching modes and δ(=CH_2_) bending modes), and in black, at the side of the UV-vis spectra, are indicated the expansion factors applied in the 450–700 nm spectral range for better data visualization.

The rupture of the H-bonds involving p2 and/or p4 dramatically affects the catalytic reaction. Heme *b* is fully formed as in the WT only in the T205V (p2) variant, confirming that in solution this residue is not H-bonded to p2 in both the coproheme and in the heme *b* complexes. Both the R208L (p2) and R139L (p4/p2) variants do not produce heme *b*, as also confirmed by the absence of the vinyl vibrations in the RR spectrum. The spectra of the R208L variant remain 6cLS, identical to those before any addition of H_2_O_2_, suggesting that the dramatic alteration of the distal cavity, which among other changes also brings the distal H118 (5.1 Å above the Fe atom in the WT [13]) to bind the sixth coordination position of the porphyrin iron, inhibits the coproheme complex from reacting with hydrogen peroxide. On the other hand, addition of hydrogen peroxide to the R208L-H118A double mutant does not cause any change in the UV-vis, second derivative, and RR spectra (Figure 5), which remain almost identical to those of the coproheme complex. Therefore, the absence of heme *b* formation in the R208L-H118A double mutant clearly indicates that the lack of the R208-p2 H-bond is the limiting step. As the propionate group p2 should be located close to the catalytic residue Y135 to allow its decarboxylation in the presence of a molecule of H_2_O_2_ [13], the rupture of the R208-p2 H-bond changes the spatial disposition of the propionate, and thus, the reaction cannot take place.

Unexpected results were obtained for the R139L variant (p4/p2) (Figure 4). Upon titration with hydrogen peroxide, the UV-Vis spectrum shows a broad Soret band at 400 nm and the presence of an intense band at 581 nm in the Q region. These peculiar features suggest the formation of iron chlorin-type heme *d*, as previously observed for the Y135A mutant, upon addition of hydrogen peroxide, or for the WT in the presence of an excess of hydrogen peroxide [22]. Heme *d* is an oxidized form of heme *b*, and its formation occurs by hydroxylation of the pyrrole ring C or D of the porphyrin macrocycle [22,23,24]. In fact, the hydroxylation of the pyrrole ring induces an out-of-plane distortion of the porphyrin ring, which determines a symmetry lowering from D_4h_ pseudosymmetry to C_2′_. This symmetry variation causes the activation of A_2g_ modes (ν_21_ and ν_20_) upon Soret excitation and an intensification of E_u_ modes (ν_41_ and ν_37_), while the intensities of the B_1g_ modes strongly decrease. Accordingly, in the RR spectrum (Figure 4, right) upon H_2_O_2_ titration, new bands at 1316, 1352 and 1389 cm^−1^ (assigned to ν_21_, ν_41_ and ν_20_, respectively) are clearly intensified, while the ν_10_ (B_1g_ mode) intensity is markedly decreased. An additional ν_3_ band at 1491 cm^−1^ is also found together with the remaining ν_3_ modes from coproheme. In the ν_2_ region, a very broad band is observed centered at 1583 cm^−1^, as a consequence of the overlapping contribution of the ν_2_ bands of the several species, together with the intensity increase of the ν_37_ (E_u_) modes of the heme *d*.

The effect of the rupture of the H-bond with p4 on heme *b* accumulation in the E113A and W143F variants reveals interesting peculiarities as well (Figure 4). The vinyl vibrations present in the RR spectra of both variants, upon addition of H_2_O_2_, confirm the heme *b* formation. However, the red-shifted UV-vis spectra (Soret at 406–408 nm and Q bands at 530–560 nm), are typical of a His-Fe-His 6cLS heme *b*-containing protein, different from the WT, which is HS (Soret band at 405 nm and Q bands at 501–537 nm). Accordingly, the RR spectra are characterized by a major 6cLS species with small amounts of 5c and 6c HS populations. These data suggest that the W143-p4 and E113-p4 H-bonds play an important role for the stability of heme *b* after rotation of the substrate within the active site.

**Figure 6:**
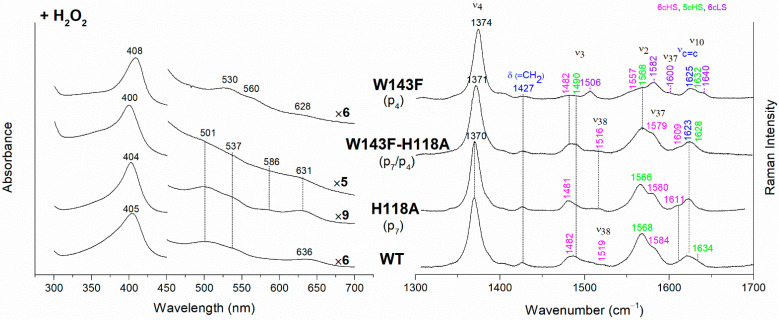
(**Left**) UV-vis electronic absorption spectra and (**Right**) RR spectra in the high wavenumber region of coproheme complexes upon H_2_O_2_ titration of *Cd*ChdC WT and H118A, W143F and H118A-W143F variants. In brackets, the propionate, whose corresponding H-bond is removed by mutation, is reported. The RR core-size marker band wavenumbers are reported in pink, green and violet for the 6cHS, 5cHS and 6cLS species, respectively, while in blue are indicated the vinyl vibrations (ν_C=C_ stretching modes and δ(=CH_2_) bending modes), and in black, at the side of the UV-vis spectra, are indicated the expansion factors applied in the 450–700 nm spectral range for better data visualization.

In their absence, a reorganization of the cavity favors the formation of the 6cLS heme *b* species. Unlike the single variant, the spectra of the W143F-H118A double mutant become similar to those of the WT (Figure 6), confirming that in the absence of the distal H118 residue, the 6cLS species cannot be formed. Therefore, the 6cLS derives from the coordination of the H118 distal residue after decarboxylation of both p2 and p4 propionates, i.e., after the rotation of the reaction intermediate MMD.

The alteration of the H-bonds involving the propionates 6 and 7 does not prevent the heme *b* formation, unless an additional steric hindrance effect occurs. In particular, as previously reported, the H118A fully forms the heme *b* complex, while the H118F produces only the MMD species since the bulky phenylalanine residue inhibits the rotation of this intermediate within the active site [11]. The T172A variant spectra resemble those of H118A (i.e., with a predominant aquo 6cHS species), whereas, analogously to the results on their coproheme complexes, the hydrogen peroxide titration of both N115L and N115R gives rise to spectra similar to that of the WT (Figure S3) with a small amount of unreacted 5cQS still present in the N115R variant.

### 3.3. Propionate H-bond Strengths

In the 360–400 cm^−1^ wavenumber region, the RR spectra are characterized by the bending modes [δ(C_β_C_c_C_d_)] of the propionate substituents of the porphyrins. It has been shown that the bending modes of heme propionates are coupled to ligand binding and heme deformation [25,26,27,28]. Their wavenumbers especially provide information on the strength of the hydrogen bonds between the propionate and the nearby polar amino acids of the protein pocket. The higher the strength of the H-bonds, the higher the bending wavenumber, and vice versa [9,28,29,30,31].

**Figure 7:**
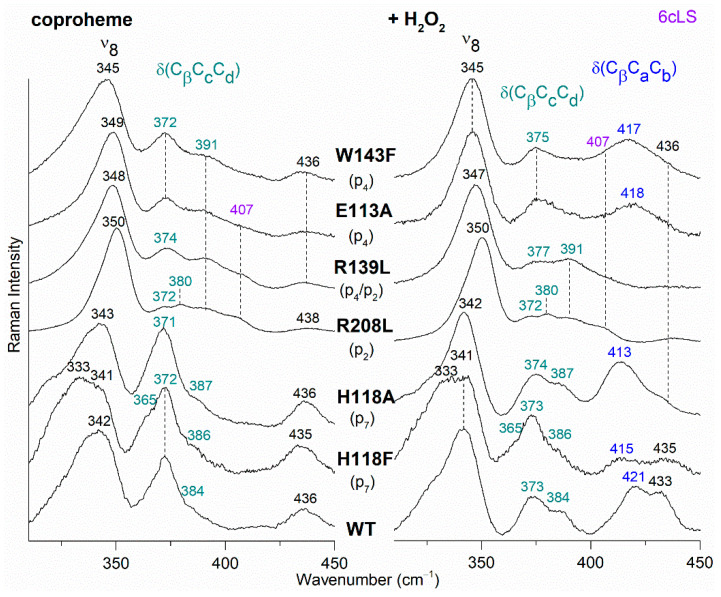
RR spectra in the low wavenumber region of WT and selected variants of *Cd*ChdC complexed with coproheme before (**Left**) and after H_2_O_2_ titration (**Right**). In brackets, the propionate, whose corresponding H-bond is removed by mutation, is reported. The band at 407 cm^−1^ due to a 6cLS species [9] is reported in violet, while the δ(C_β_C_c_C_d_) propionate and the δ(C_β_C_a_C_b_) vinyl bending modes are in green and blue, respectively.

The RR spectra of coproheme complexes of selected variants of *Cd*ChdC, which display a change in the propionate bending mode region before (left panel) and after (right panel) titration with hydrogen peroxide, are reported in Figure 7 in comparison with the reconstituted WT heme *b* complex. It is worth noting that no spectral differences were observed for T205V and N115L variants in the low wavenumber region with respect to the WT, while N115R variant showed only small intensity changes of the propionate bending modes and no modifications in their wavenumbers (data not shown). The relative intensity of the propionate bending modes and their correlation with that of the other bands in the low wavenumber region (i.e., ν_9_, γ_9_, ν_8_) is still not rationalized [30]. Since these variants (T205V, N115L, N115R) also exhibit UV-Vis and core size RR bands very similar to the WT, we might infer that the H-bonds involving the propionate with these residues are not maintained in the solution or are not relevant for the stability of the porphyrin group inside the pocket.

**Figure 8:**
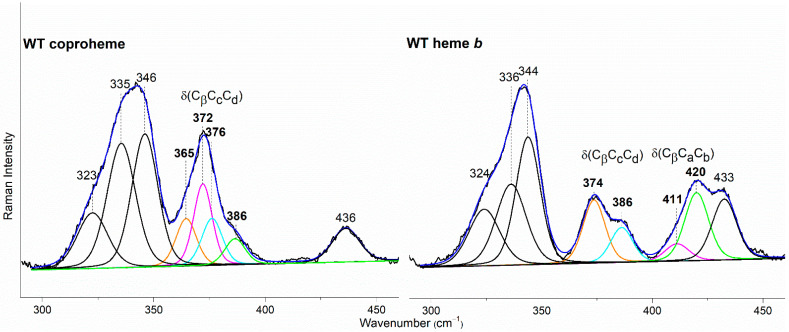
Curve-fitting analysis of the RR spectra of *Cd*ChdC WT in complex with coproheme (**Left**) and heme *b* (**Right**). The bands indicated in black at 323, 335, 346, and 436 cm^−1^ in the coproheme complex and at 324, 336, 344, and 433 cm^−1^ in the heme *b* complex, are assigned to the ν_17_, γ_6,_ ν_8_, and γ_22_/δ (C_β_-Me) porphyrin modes, respectively, according to Refs. [32,33,34]. In the WT coproheme complex, the bands indicated in bold at 365, 372, 376, and 386 cm^−1^ are assigned to p7 (orange), p2 (magenta), p6 (cyan), and p4 (green) propionate bending modes (see text), respectively. In the WT heme *b* complex the bands indicated in bold at 374 and 386 cm^−1^ are assigned to p7 (orange) and p6, (cyan) respectively, while the bands at 411 and 420 cm^−1^ are assigned to the vinyl bending modes in position v2 (magenta) and v4 (green), respectively [10,11]. The corresponding bandwidths are 11 and 13 cm^−1^ for the propionate and vinyl bending modes, respectively, while they are within the 13–17 cm^−1^ range for the other porphyrin bands.

In the WT coproheme complex, the four propionate bending modes are overlapped (Figure 7 left, bottom spectrum) and a curve-fitting analysis evidences the presence of four bands at 365, 372, 376, and 386 cm^−1^ (Figure 8). The analysis of the RR spectra of the variants in the low wavenumber region in principle should allow one to selectively assign the corresponding vibrational bending modes. In fact, the rupture (or the weakening) of the H-bond between the mutated apolar residue and the propionate group should result mainly in a decrease in the wavenumber of the corresponding bending vibration. However, due to the presence of slightly different H-bond distances between the propionyls and the residues in the five subunits of the protein (see Table 1) and to the extensive H-bond network spanning from p2 to p4 (see Figure 1), it is impossible to predict the net effect that single or double mutations could exert on the wavenumber of each propionate bending mode.

The RR spectrum of the H118F coproheme complex shows three bands at 365, 372 and 386 cm^−1^ (Figure 7, left), and as for the WT, the deconvolution of this spectral region results in four bands at 363, 372, 376 and 386 cm^−1^ (Figure S4) [32]. The lowest wavenumber bending mode, downshifted by 2 cm^−1^ as compared to the WT (Figure 8, left), is assigned to the propionate group p7, since in the H118F variant the H-bond with this propionate is lost. Titration of the H118F coproheme complex with H_2_O_2_ gives rise to an RR spectrum very similar to that of its coproheme complex, but with a decreased intensity of the [δ(C_β_C_c_C_d_)] bands with respect to the ν_8_ at 341 cm^−1^ and with a new band at 415 cm^−1^ assigned to a δ(C_β_C_a_C_b_) vinyl bending mode (Figure 7, right). The deconvoluted spectrum (Figure S4) shows the presence of four propionate bending modes at the same wavenumber of the coproheme complex (363, 372, 376, and 386 cm^−1^) and confirms the intensity weakening of the bands at 363 and 372 cm^−1^. Mass spectrometry studies have shown that by adding hydrogen peroxide, the H118F mutant does not completely react, as the substrate cannot rotate within the active site pocket. As a consequence, together with the unreacted coproheme complex, only the MMD intermediate is observed, characterized by a vinyl in position 2 and three propionates [11]. Therefore, since only p2 is decarboxylated, the decreased intensity of the band at 372 cm^−1^ upon hydrogen peroxide titration enables its assignment to the p2 bending mode in both the WT and H118F coproheme complexes. The concomitant decrease in the p7 band intensity (Figure 7, right, and Figure S4) suggests that the absence (or weakening) of the H-bonds with p2 destabilizes also the H-bond with p7. This is consistent with the results obtained for the R208L variant, whose H-bond with p2 is removed by the mutation. This mutation causes an overall intensity decrease in the propionate bending modes, and only three bands can be distinguished at 372, 380, and 391 cm^−1^. Since p2 is H-bonded not only to R208, but also to R139, which in turn is H-bonded to p4, the observed changes suggest that the band at 380 cm^−1^ derives from the strengthening of the H-bond involving the p2, while the band at 391 cm^−1^ is assigned to the bending mode involving the p4 (at 386 cm^−1^ in the deconvolution of the WT spectrum). In fact, the band at 391 cm^−1^ is observed in all of the other studied mutants involving an H-bond interaction with p4, namely R139L, E113A and W143F. Interestingly, both p2 and p4 H-bonds appear to be strengthened after mutation, probably resulting from the readjustment of the other H-bonds involving either p2 or p4 (See Figure 1 and Table 1). Thus, the remaining bending mode at 376 cm^−1^ observed in the deconvoluted spectrum of WT is assigned, by exclusion, to the p6 bending mode.

**Figure 9:**
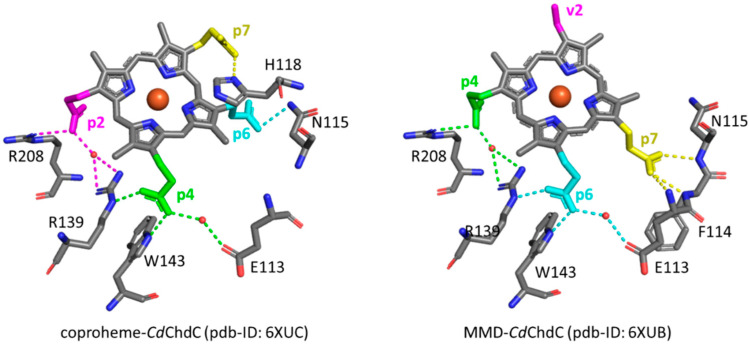
Hydrogen bonding interactions (dotted lines) of propionate groups in coproheme and 90° rotated MMD *Cd*ChdC complexes. Dotted lines of the p2 H-bonding interactions are depicted in magenta, p4 in green, p6 in cyan, and p7 in yellow, respectively. All other carbon atoms are represented in grey, oxygen atoms in red, nitrogen atoms in blue and the heme iron in orange [13]. Red circles indicate water molecules.

The selective assignment of the propionate bending modes allows us to rationalize the changes observed in the low wavenumber region upon formation of heme *b*. Titration with hydrogen peroxide results in the decarboxylation of both p2 and p4 into vinyl groups, and the final product, heme *b*, is rotated clockwise by 90° compared to the coproheme complex structure. As a consequence, in the RR low wavenumber region of the heme *b* complexes, the bending modes [δ(C_β_C_a_C_b_)] of the vinyl groups, v2 and v4, and [δ(C_β_C_c_C_d_)] of two propionates, p6 and p7, are detected. Due to the rotation, p6 moves to the original position of p4 in the coproheme structure, and p7 moves to the former p6 position. Accordingly, the p6 bending mode wavenumber upshifts from 376 cm^−1^ (WT coproheme complex) to 386 cm^−1^ (WT heme *b* complex), and p7 from 365 to 374 cm^−1^ (Table 2). Therefore, the bending mode wavenumbers of p6 and p7 in the heme *b* complex are very close to those of p4 and p6 before rotation, respectively, indicating that p6 and p7 after rotation are involved in interaction with a chemical environment very similar to that of p4 and p6 before the titration with H_2_O_2_. The environment of p4 in coproheme and p6 in MMD complexes is identical, whereas the p7 interactions change from one H-bond to H118 to interactions with the backbone nitrogens of E113, F114, and N115. As these H-bonds of p7 after the first decarboxylation and rotation of MMD are formed with backbone atoms, they are insensitive to mutations (Figure 9). A similar result has been previously observed for *Lm*ChdC [9,35].

## 4. Discussion

Our results confirm that the extensive H-bond network formed by polar residues of the protein pocket and the propionate groups of the coproheme, as observed by the X-ray structure, is maintained in solution. The only exception is represented by the H-bond between T205 and p2, which is only formed in the crystal (Figure 1 and Table 1). Among all, the H-bond interactions involving the propionates in position 2 and 4 are essential for the stability of the porphyrin ring inside the pocket and for keeping the spatial disposition to allow their decarboxylation by the Y135 catalytic residue. In the coproheme complex, the mutation of the amino acid residue R208 (R208L) leads to the formation of a single 6cLS species. Therefore, the loss of the interaction with the p2 group leads to a collapse of the protein cavity, directing the distal H118 residue (located about 5 Å above the heme iron in the protein–coproheme structure [13]) to bind the Fe atom. This variant is unable to react with hydrogen peroxide, not only because the heme iron is strongly bound to the distal H118 residue and, therefore, prevents its ability to deprotonate incoming H_2_O_2_, but also because the lack of the R208-p2 H-bond changes the p2 correct spatial disposition to be decarboxylated by Y135. In fact, the double mutant R208L-H118A becomes mainly a high spin species due to the lack of the distal His, but is still unable to react with hydrogen peroxide.

The mutation of the R139 residue (R139L), leads to the formation of both 6cHS and 6cLS species at the expense of the 5cHS and 5cQS species of the WT. The presence of multiple species following the mutation suggests an increased mobility of the active site pocket as compared to the WT, due to the simultaneous rupture of both the H-bonds with p2 and p4. This variant is also inactive and forms heme *d* in the presence of hydrogen peroxide. The single E113A mutation gives rise to an effect similar, but to a lesser extent, to that of the R139L variant, since the coproheme complex shows the presence of both high and low spin species. On the contrary, mutation of the W143 causes only minor spectral changes in the coproheme complex since the rupture of its p4 H-bond does not alter the remaining R139 and E113 H-bond interactions. However, both W143F and E113A variants do react with hydrogen peroxide, but the product in this case is a 6cLS heme *b*. For the W143F variant, we have identified the distal H118 residue as the internal ligand of the heme iron. The fact that this bis-histidine complex is formed upon 90° rotation of the porphyrin clearly indicates that the W143-p4 H-bond contributes to the maintenance of the integrity of the distal heme pocket after rotation of the MMD reaction intermediate.

Mutations on the p6 and p7 do not alter either the porphyrin ring stability within the cavity or the reactivity with hydrogen peroxide. The major result concerning these systems is the steric hindrance by the bulky phenylalanine residue in the H118F variant that blocks the rotation of the MMD intermediate, preventing the formation of heme *b*, as already reported [11]. On the contrary, the H118A mutation does not alter heme *b* accumulation upon hydrogen peroxide titration [11,13]. Interestingly, the displacement of H118, which is specific for actinobacterial ChdCs [36], as obtained by breaking the H-bond with T172 (T172A) results in an effect similar to that from completely removing the residue (e.g., in the H118A variant).

The mutations allowed us to selectively assign the bending modes of all four propionyl substituents of coproheme. The H-bonds between p2, p6 and p7 with the amino acids of the cavity are preserved with similar strength, as suggested by the propionate bending mode wavenumbers, which in the WT–coproheme complex give rise to an intense broad band centered at 372 cm^−1^. On the contrary, p4, being involved in multiple H-bonds with both the terminal oxygen atoms (Figure 1), results in an upshift of its bending mode wavenumber, as compared to other propionates.

Based on the assignment, we could conclude that the p6 and p7 bending modes (present in both coproheme and heme *b*) upshift by about 10–12 cm^−1^ in WT heme *b Cd*ChdC WT complex as compared to coproheme *Cd*ChdC. In agreement with the 90° clockwise rotated pose of heme *b* with respect to that of coproheme, the data confirm a change in the H-bonding interactions as going from the substrate to the product. In this latter configuration, p6 interacts with W143, E113, and R139 (formerly H-bonded to p4), while p7 interacts with the backbone nitrogen atoms of N115 (formerly H-bonded to p6) of E113 and F114 (Figure 8). Accordingly, all of the mutants, which form heme *b* upon titration with hydrogen peroxide, give rise to very similar spectra, characterized by a high spin heme *b* with propionate and vinyl bending modes that do not markedly differ from the WT heme *b*. The only exceptions are represented by the W143F and E113A variants, where the product is a 6cLS heme *b* (bis-histidine type with the distal H118 bound to the heme iron), with a very weak p6 bending mode. Clearly, the H-bond interactions with W143 and E113 are fundamental for the stability of heme *b* in the distal cavity.

## 5. Conclusions

In this work, we systematically dissected the complex and highly specific H-bonding network of actinobacterial ChdCs. This now describes the active site architecture at the molecular level in very high detail and highlights the importance of each protein–coproheme/MMD/heme *b* interaction. This enables us to assign specific roles to the residues of the protein pocket, interacting with the propionate groups of the porphyrin, and also to the distal histidine. This distal histidine is part of a flexible loop, connecting the N-terminal and C-terminal domain of one ChdC subunit, and is specific for actinobacterial representatives [36]. This work substantially helps in the process of defining ChdCs as a suitable druggable target for future studies. Heme biosynthesis is crucial for the survival of many actinobacterial pathogens, e.g., *Corynebacterium diphtheria* or *Mycobacterium tuberculosis* [37,38], and thus a rational inhibitor design for ChdC will only be possible with a profound knowledge of the active site architecture and of the factors contributing to substrate stability.

## Figures and Tables

**Table 1:** Hydrogen bond distances (in Å) between the polar residues and the coproheme propionate groups in the active site of the five chains of *Cd*ChdC, as evinced by the crystal structure (PDB ID: 6XUC) [13].

	Chain A	Chain B	Chain C	Chain D	Chain E
**R208**	3.0-p2	3.2-p2	3.0-p2	3.0-p2	3.1-p2
**T205**	2.6-H_2_O-2.5-p2	2.7-H_2_O-2.8-p2	2.7-H_2_O-2.7-p2	2.8-H_2_O-2.6-p2	2.7-H_2_O-2.6-p2
**R139**	2.9-H_2_O-2.5-p2	2.8-H_2_O-2.5-p2	3.0-H_2_O-2.6-p2	2.9-H_2_O-2.7-p2	3.1-H_2_O-2.5-p2
**R139**	2.7-p4	2.8-p4	2.7-p4	2.6-p4	2.7-p4
**W143**	2.8-p4	2.8-p4	2.7-p4	2.7-p4	2.7-p4
**E113**	2.7-H_2_O-2.5-p4	3.2-H_2_O-2.4-p4	2.9-H_2_O-2.5-p4	2.9-H_2_O-2.7-p4	3.6-H_2_O-3.8-p4
**N115**	3.2-p6	3.2-p6	3.1-p6	3.2-p6	3.5-p6
**H118**	2.8-p7	3.0-p7	2.6-p7	2.9-p7	3.2-p7
**T172**	2.7-H118-2.8-p7	2.7-H118-3.0-p7	2.7-H118-2.6-p7	2.8-H118-2.9-p7	3.1-H118-3.2-p7

**Table 2:** Propionate bending mode wavenumbers (cm^−1^), as observed in the RR spectra or according to the deconvoluted spectra (reported in italics in brackets) of WT and variants (see Figure 8 and Figure S3).

	p7	p2	p6	p4
	**coproheme**			
**WT**	-- (*365*)	372 (*372*)	---- (*376*)	384 (*386*)
**H118F (p7)**	365 (*363*)	372 (*372*)	---- (*376*)	386 (*386*)
**H118A (p7)**		371		387
**W143F (p4)**		372		391
**E113A (p4)**		372		391
**R139L (p4/p2)**		374		391
**R208L (p2)**		380	372	391
	**+H_2_O_2_**			
**WT (heme *b*)**	373 (*374*)		384 (*386*)	
**H118F (MMD)**	365 (*363*)	373 (*372*)	---- (*376*)	386 (*386*)
**H118A (heme *b*)**	374		387	
**W143F (heme *b*)**	375		weak	
**E113A (heme *b*)**	375		weak	

## Data Availability

Not applicable.

## Supplementary Materials

The following supporting information can be downloaded at: https://www.mdpi.com/article/10.3390/biom13020235/s1, Figure S1: Structures of coproheme, MMD and heme *b*; Figure S2: Comparison of the electronic absorption and RR spectra of coproheme *Cd*ChdC complexes of WT and single variants involving the H-bond interactions with p6 and p7; Figure S3: Comparison of the electronic absorption and RR spectra of coproheme *Cd*ChdC complexes of WT and single variants involving the H-bond interactions with p6 and p7 upon titration with H_2_O_2_; Figure S4: Curve fitting analysis of the RR spectra of H118F coproheme and MMD complexes; Table S1: Primers used for site-directed mutagenesis to create variants of *Cd*ChdC (vector: pD441-NH); Table S2: Experimental conditions used to obtain the reported RR spectra.
