# Supplementary material for: The Role of the Hydrogen Bond Network in Maintaining Heme Pocket Stability and Protein Function Specificity of C. diphtheriae Coproheme Decarboxylase
Source: Biomolecules. 2023 Jan 25;13(2):235. doi: 10.3390/biom13020235 (PMC9953210; doi:10.3390/biom13020235)
Supplement: Supplementary file 1 [file biomolecules-13-00235-s001.zip › biomolecules-2146397-supplementary.pdf]

Supplemental Information

The role of the hydrogen bond network in maintaining heme pocket stability and protein function specificity of *C. diphtheriae* coproheme decarboxylase.

Federico Sebastiani[1], Chiara Baroni[1], Gaurav Patil[2], Andrea Dali[1], Maurizio Becucci[1]*, Stefan Hofbauer[2]*, and Giulietta Smulevich[1,3]*

[1]*Dipartimento di Chimica "Ugo Schiff", DICUS, Università di Firenze, Via della Lastruccia 3-13, I-50019 Sesto Fiorentino (FI), Italy*

[2]*Department of Chemistry, Institute of Biochemistry, University of Natural Resources and Life Sciences, Muthgasse 18, A-1190 Vienna, Austria*

[3]*INSTM Research Unit of Firenze, via della Lastruccia 3, I-50019 Sesto Fiorentino, Italy*

KEYWORDS: prokaryotic heme biosynthesis, resonance Raman, heme b, propionate bending modes, variants.

Running Title: Active site architecture of Cd*ChdC*

Corresponding Authors

*e-mail: Maurizio.becucci@unifi.it, phone: +39-055-4573089

*e-mail: stefan.hofbauer@boku.ac.at, phone: +43-1-47654-77258, fax: +43-1-47654-77059

*e-mail: giulietta.smulevich@unifi.it, phone: +39-055-4573083

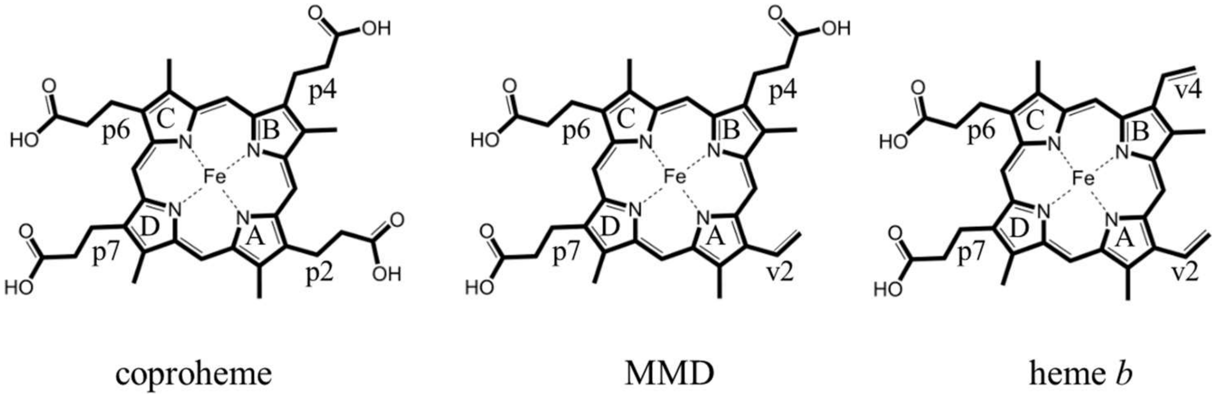

Figure S1 Structures of coproheme, MMD and heme *b*. The peripheral groups and pyrrole rings have been labelled.

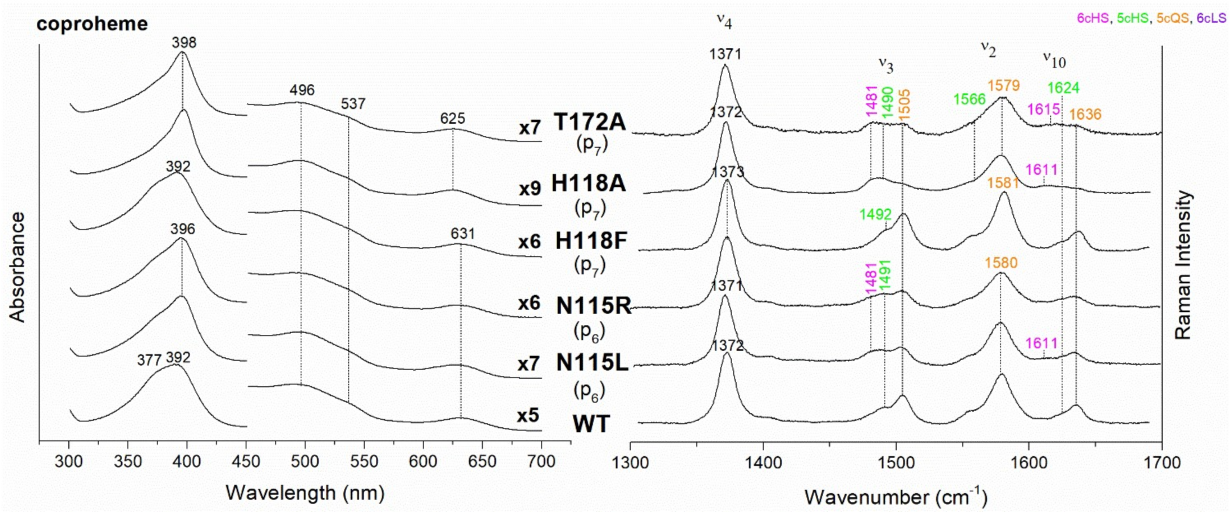

Figure S2 (Left) UV-vis electronic absorption spectra and (Right) RR spectra in the high wavenumber region of coproheme complexes of *Cd*ChdC WT and variants. In brackets the propionate, whose corresponding H-bond is removed by mutation, is reported. The RR core-size marker band wavenumbers are reported in pink, green, orange and violet for the 6cHS, 5cHS, 5cQS and 6cLS species, respectively. In black, at the side of the UV-vis spectra, are indicated the expansion factors applied in the 450-700 nm spectral range for better data visualization.

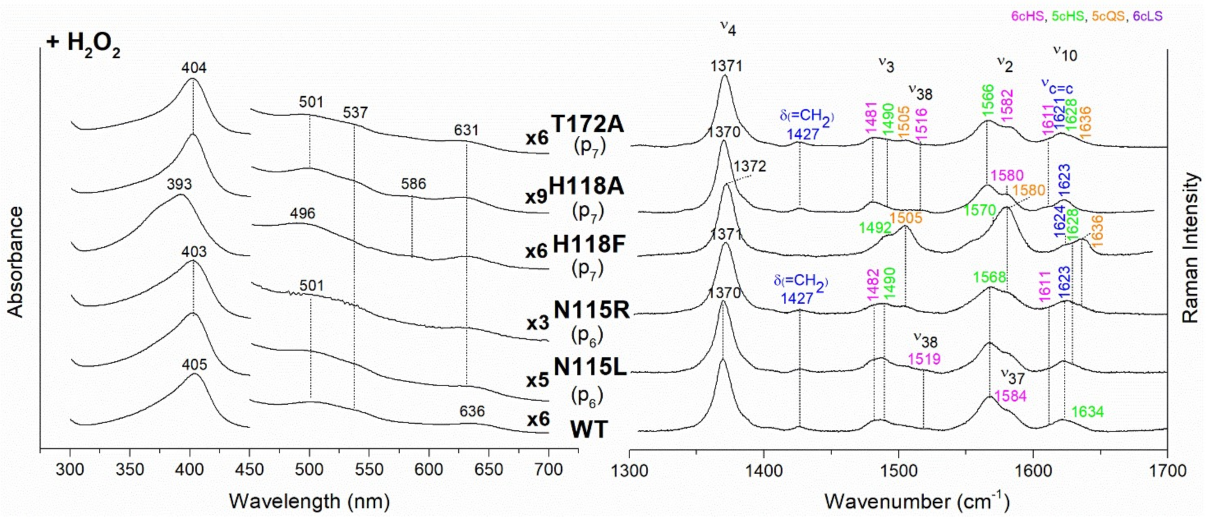

Figure S3 (Left) UV-vis electronic absorption spectra and (Right) RR spectra in the high wavenumber region of coproheme complexes upon H_2O_2 titration of *Cd*ChdC WT and variants complexes. In brackets the propionate, whose corresponding H-bond is removed by mutation, is reported. The RR core-size marker band wavenumbers are reported in pink, green, orange and violet for the 6cHS, 5cHS, 5cQS and 6cLS species, respectively. While in blue are indicated the vinyl vibrations ($\nu_{C=C}$ stretching modes and δ(=CH_2) bending modes) and in black, at the side of the spectrum, are indicated the expansion factors applied in the 450-700 nm spectral range for better data visualization.

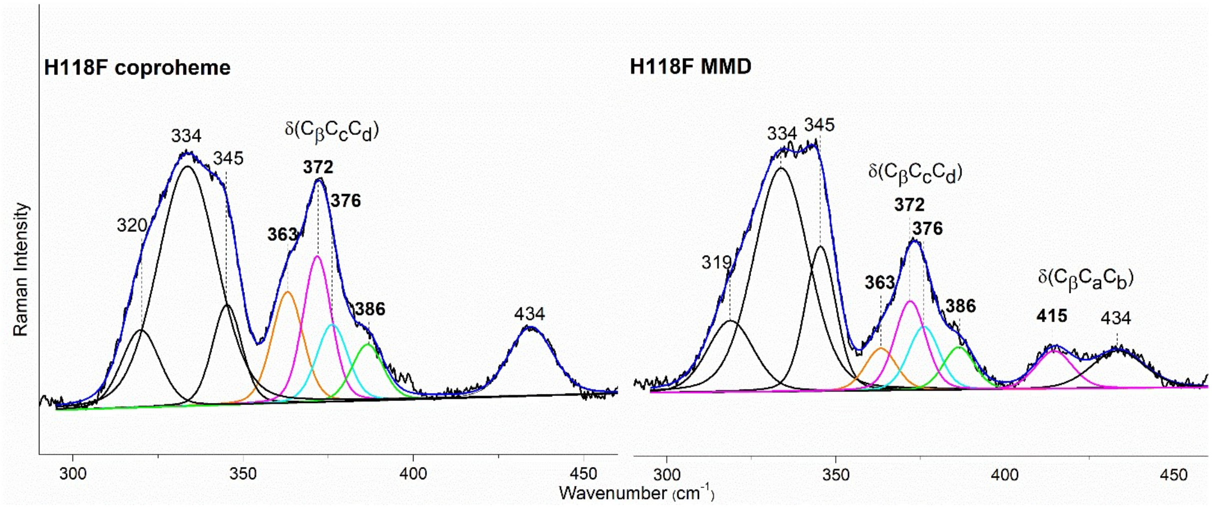

Figure S4: Curve-fitting analysis of the RR spectra of *Cd*ChdC H118F in complex with coproheme (left) and MMD (right). The bands indicated in black at 320, 334, 345, and 434 cm^{-1} in the coproheme complex and at 319, 334, 345, and 434 cm^{-1} in the MMD complex, are assigned to the ν_{17}, γ_{6}, ν_{8}, γ_{22}/δ(C_{β}-Me) porphyrin modes, respectively, according to Refs [1-3]. In the H118F coproheme and MMD complexes the bands at 363, 372, 376, 386 cm^{-1} are assigned to p7, p2, p6, and p4 propionate bending modes (see main text), respectively. In the H118F MMD complex the band at 415 cm^{-1} is assigned to the vinyl bending modes in position 2 [4]. The corresponding bandwidths are 11 and 13 cm^{-1} for the propionate and vinyl bending modes, respectively, while are within the 11-21 cm^{-1} range for the other porphyrin bands.

Table S1. Primers used for site-directed mutagenesis to create variants of *Cd*ChdC (vector: pD441-NH).

Mutation	
E113A	catcgcccggctgcgttcaaccgcagc<br>gctgcggttgaacgcagccgggcgatg
N115L	cgcccggctgagttcctacgcagccatctgccg<br>cggcagatggctgcgtaggaactcagccgggcg
N115R	gatggctgcgcctgaactcagccgggcgat<br>atcgcccggctgagttcaggcgcagccatc
H118A	ctgagttcaaccgcagcgctctgccgtcgttcatta<br>taatgaacgacggcagagcgctgcggttgaactcag
H118F	gctgagttcaaccgcagctttctgccgtcgttcattat<br>ataatgaacgacggcagaaagctgcggttgaactcagc
R139L	ggtttacccgttcgtgctcagctacgattggtata<br>tataccaatcgtagctgagcacgaacgggtaaacc
W143F	tcgtgcgcagctacgatttctatatcatggacccacag<br>ctgtgggtccatgatatagaaatcgtagctgcgcacga
T172A	ctgacgtccgtgcaaatgccgtgccgg<br>ccggcacggcatttgcacggacgtcag
T205V	gatctgatgcacaagatgcgttatgtcgaagcccgcctg<br>caggcgggcttcgacataacgcatcttgtgcatcagatc
A207E	gaacgtgcaggcgctcttcggtataac<br>cgttataccgaagagcgcctgcacgtt
R208L	cgcgaacgtgcaggagggcttcggtataa<br>ttataccgaagccctcctgcacgttcgcg

Table S2. Summary of the integration time (in minutes) and number of averaged RR spectra as obtained upon 404.8 nm laser excitation and reported in this work for the coproheme complexes before and after H_2O_2 titration of the *Cd*ChdC investigated variants. The spectra of the WT, H118A and H118F proteins are obtained upon 406.7 nm laser excitation and reproduced from Refs.[4,5].

Variant		High Wavenumber (average/ integration time)	Low Wavenumber (average/ integration time)
T205V	coproheme	24 spectra/120 min.	48 spectra/240 min.
	+ H_2O_2	24 spectra/120 min.	36 spectra /180 min.
R208L	coproheme	18 spectra/90 min.	36 spectra/180 min.
	+ H_2O_2	12 spectra/60 min.	12 spectra/60 min.
R139L	coproheme	16 spectra/80 min.	28 spectra/140 min.
	+ H_2O_2	16 spectra/80 min.	20 spectra/100 min.
W143F	coproheme	24 spectra/120 min.	36 spectra/180 min.
	+ H_2O_2	18 spectra/90 min.	36 spectra/180 min.
E113A	coproheme	16 spectra/80 min.	20 spectra/100 min.
	+ H_2O_2	24 spectra/120 min.	32 spectra/160 min.
N115L	coproheme	20 spettri/100 min.	24 spettri/120 min.
	+ H_2O_2	28 spectra/140 min.	44 spectra 220 min.
N115R	coproheme	24 spectra/120 min.	36 spectra/180 min.
	+ H_2O_2	18 spectra/90 min.	24 spectra/120 min.
W143F-H118A	coproheme	14 spectra/70 min.	16 spectra/80 min.
	+ H_2O_2	12 spectra/60 min.	24 spectra/120 min.
R208L-H118A	coproheme	24 spectra/120 min.	36 spectra/180 min.
	+ H_2O_2	12 spectra/60 min.	12 spectra/60 min.
T172A	coproheme	8 spectra/40 min.	20 spectra/100 min.
	+ H_2O_2	20 spectra/100 min.	24 spectra/120 min.

1. Brogioni, S.; Feis, A.; Marzocchi, M.P.; Zederbauer, M.; Furtmuller, P.G.; Obinger, C.; Smulevich, G. Resonance Raman assignment of myeloperoxidase and the selected mutants Asp94Val and Met243Thr. Effect of the heme distortion. *J Raman Spectrosc* **2006**, *37*, 263-276.
2. Smulevich, G.; Hu, S.; Rodgers, K.; Goodin, D.; Smith, K.; Spiro, T. Heme-protein interactions in cytochrome c peroxidase revealed by site-directed mutagenesis and resonance Raman spectra of isotopically labeled hemes. *Biospectroscopy* **1996**, *2*, 365-376, doi:10.1002/(SICI)1520-6343(1996)2:6<365::AID-BSPY3>3.0.CO;2-2.
3. Hu, S.Z.; Smith, K.M.; Spiro, T.G. Assignment of protoheme Resonance Raman spectrum by heme labeling in myoglobin. *J Am Chem Soc* **1996**, *118*, 12638-12646.
4. Sebastiani, F.; Michlits, H.; Lier, B.; Becucci, M.; Furtmüller, P.G.; Oostenbrink, C.; Obinger, C.; Hofbauer, S.; Smulevich, G. Reaction intermediate rotation during the decarboxylation of coproheme to heme b in C. diphtheriae. *Biophys J* **2021**, *120*, 3600-3614, doi:10.1016/j.bpj.2021.06.042.
5. Sebastiani, F.; Risorti, R.; Niccoli, C.; Michlits, H.; Becucci, M.; Hofbauer, S.; Smulevich, G. An active site at work - the role of key residues in C. diphteriae coproheme decarboxylase. *J Inorg Biochem* **2022**, *229*, 111718, doi:10.1016/j.jinorgbio.2022.111718.
